# Reconstructing the phylogeny of 21 completely sequenced arthropod species based on their motor proteins

**DOI:** 10.1186/1471-2164-10-173

**Published:** 2009-04-21

**Authors:** Florian Odronitz, Sebastian Becker, Martin Kollmar

**Affiliations:** 1 Department of NMR-based Structural Biology, Max-Planck-Institute for Biophysical Chemistry, Am Fassberg 11, 37077 Goettingen, Germany

## Abstract

**Background:**

Motor proteins have extensively been studied in the past and consist of large superfamilies. They are involved in diverse processes like cell division, cellular transport, neuronal transport processes, or muscle contraction, to name a few. Vertebrates contain up to 60 myosins and about the same number of kinesins that are spread over more than a dozen distinct classes.

**Results:**

Here, we present the comparative genomic analysis of the motor protein repertoire of 21 completely sequenced arthropod species using the owl limpet *Lottia gigantea *as outgroup. Arthropods contain up to 17 myosins grouped into 13 classes. The myosins are in almost all cases clear paralogs, and thus the evolution of the arthropod myosin inventory is mainly determined by gene losses. Arthropod species contain up to 29 kinesins spread over 13 classes. In contrast to the myosins, the evolution of the arthropod kinesin inventory is not only determined by gene losses but also by many subtaxon-specific and species-specific gene duplications. All arthropods contain each of the subunits of the cytoplasmic dynein/dynactin complex. Except for the dynein light chains and the p150 dynactin subunit they contain single gene copies of the other subunits. Especially the roadblock light chain repertoire is very species-specific.

**Conclusion:**

All 21 completely sequenced arthropods, including the twelve sequenced *Drosophila *species, contain a species-specific set of motor proteins. The phylogenetic analysis of all genes as well as the protein repertoire placed *Daphnia pulex *closest to the root of the Arthropoda. The louse *Pediculus humanus corporis *is the closest relative to *Daphnia *followed by the group of the honeybee *Apis mellifera *and the jewel wasp *Nasonia vitripenni*s. After this group the rust-red flour beetle *Tribolium castaneum *and the silkworm *Bombyx mori *diverged very closely from the lineage leading to the *Drosophila *species.

## Background

Nearly each single cell in eukaryotes hosts particular proteins, which are responsible for intracellular transport. These molecular motor molecules are highly conserved among the different species of eukaryotes and evolved slowly over time [1,2]. This property grants them the role of an appropriate candidate to carry out evolutionary studies. The three superfamilies of transporting motor proteins are the myosins, kinesins, and dyneins. Attached to the cytoskeletal networks (microtubules and actin) they transport all kinds of organelles and vesicles [3], and organize and remodel the cytoskeleton and developmental processes in eukaryotes [4]. The energy for their unidirectional cargo transport on one of the filamentous cytoskeletal tracks is derived from ATP hydrolysis [5]. Out of the three superfamilies only the members of the kinesin superfamily are found in all eukaryotes, whereas members of the dynein [6] and myosin [7] superfamilies are lacking in particular eukaryotic lineages.

The members of the actin-based myosin family have their origin early in eukaryotic evolution. Based on the latest analysis, the myosins are grouped into 35 classes [7]. Myosins consist of three regions, the motor (or head) domain, a neck domain, and the tail, which comprises all C-terminal domains as well as domains N-terminal to the motor domain. The motor domain is highly conserved and contains both the ATP and actin binding site, where the force generation resides. This energy-transducing motor domain is coupled to a regulatory neck region (helical region), which is able to bind calmodulin or calmodulin-like light chains. Linked to the neck region most myosins have tail domains. Contrary to the head domains the tail domains show high variability in sequence and length, reflecting their functional diversity. The functions range from cytokinesis, organellar transport, cell polarization to signal transduction [8-10]. Some of the myosin classes also contain large domains at the N-terminus of the motor domains [7].

The second molecular motor protein family is kinesin (members also known as KRPs, KLPs, or KIFs) [11]. The members of this superfamily are microtubule-based and facilitate movement in both directions (either plus or minus end-directed) [12]. For their movement along the microtubules they utilize ATP similarly to the other motor proteins. The classical kinesin forms a tetramer with two kinesin heavy chains (KHCs) and two kinesin light chains (KLCs). Like in myosins the head domain is well conserved and responsible for the movement, whereas the stalk and tail domains play fundamental roles in the interaction with other subunits of the holoenzyme or with cargo molecules such as proteins, lipids or nucleic acids [13]. The region between the head and the stalk is family-specific and determines the direction of movement [14]. Kinesins bind a variety of cargoes and perform tasks such as vesicle and organelle transport, spindle formation and elongation, chromosome segregation, and microtubule organization [15,16].

The members of the dynein superfamily are minus end-directed motor proteins [17]. Thus they are responsible for the retrograde transport of cargoes along microtubules. They are involved in many processes like spindle formation, chromosome segregation, and the transport of a variety of cargoes like viruses, RNAs, signaling molecules, and organelles [18]. Dyneins are multi-subunit protein complexes with two or three heavy chains (DHCs), light chains, light intermediate, and intermediate chains [19]. Supported by an activator protein called dynactin, which consists of 11 subunits, dynein is able to move and bind to membranes or further cargoes [20-22].

The genome of *Drosophila melanogaster *was the third eukaryotic genome to be completely sequenced [23]. Since then, the number of sequenced organisms has increased rapidly. Of the Arthropoda phylum, the genomes of the mosquitos *Anopheles gambiae *[24] and *Aedes aegyptii *[25], the silkworm *Bombyx mori *[26,27], the beetle *Tribolium castaneum *[28], the waterflea *Daphnia pulex *(this special series in BMC journals), and eleven of the *Drosophila *species group [29,30] have been published. The draft genome sequences of *Culex pipiens quinquefasciatus*, *Nasonia vitripennis*, and *Pediculus humanus corporis *have been finished recently. The phylogenetic relationship of the twelve sequenced *Drosophila *species has been described in detail [29].

Here, we present the analysis of the phylogenetic relationship of 21 completely sequenced arthropods based on the sequences and inventory of their motor proteins.

## Results

### Identification and annotation of the motor proteins

The arthropod motor protein genes were identified by TBLASTN searches against the corresponding genome data of the different species. Species, that missed certain orthologs in the first instance, were searched again with supposed-to-be orthologs of the other species. In this iterative process all motor proteins have been identified or their absence in certain species have been confirmed. The species analyzed were the mosquitos *Aedes aegyptii *(*Aea*), *Culex pipiens quinquefasciatus *(*Cpq*), and *Anopheles gambiae *(*Ang*), the silkworm *Bombyx mori *(*Bm_b*), the honeybee *Apis mellifera *(*Am*), the jewel wasp *Nasonia vitripennis *(*Nav*), the waterflea *Daphnia pulex *(*Dap*), the rust-red flour beetle *Tribolium castaneum *(*Tic*), the body louse *Pediculus humanus corporis *(*Pdc*), twelve *Drosophila *species (*Drosophila ananassae *(*Da*), *Drosophila erecta *(*Der*), *Drosophila grimshawi *(*Dg*), *Drosophila melanogaster *(*Dm*), *Drosophila mojavensis *(*Dmo*), *Drosophila persimilis *(*Dp*), *Drosophila pseudoobscura *(*Drp*), *Drosophila sechellia *(*Dse*), *Drosophila simulans *(*Dss_a*), *Drosophila virilis *(*Dv*), *Drosophila willistoni *(*Dw*) and *Drosophila yakuba *(*Dy*)), and the mollusc *Lottia gigantea *(*Lg*), which we used as outgroup. The sequences were assigned by manual inspection of the genomic DNA sequences. Exons have been confirmed by the identification of flanking consensus intron-exon splice junction donor and acceptor sequences [31]. The genomic sequences of *Drosophila virilis*, *Apis mellifera*, and especially *Bombyx mori *contain several gaps. Many of the gaps have been filled by analyzing EST data.

### Analysis of the arthropod myosins

**Figure 1:**
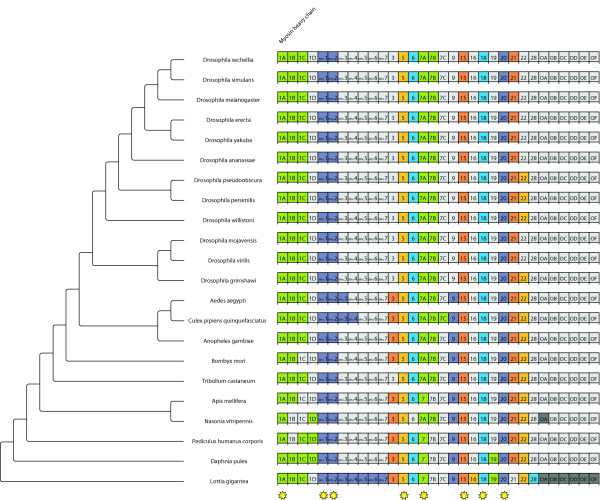
**Myosin repertoire of the arthropods**. This chart shows the myosin repertoire for all species in the analysis. To the left is a schematic phylogenetic tree, depicting the relationships (no scale). The identifiers in the boxes indicate protein classes/variants. "O" means orphan class. Colored boxes mean the class/variant exists in this species. Grey boxes mean the class/variant was not found. Columns marked with stars were included in the phylogenomic analysis.

All myosins have been classified based on the phylogenetic analysis of their motor domains together with the motor domains of the already grouped myosins [7] (Figure 1). All myosins belong to previously defined classes except one myosin from *Nasonia *that has a very similar domain organization to the class-V myosins but a considerably different motor domain. Except for class-XXI, all myosin classes are shared between arthropods and mammals suggesting that their common ancestor already contained these classes [7]. *Daphnia*, which roots the insect phylogeny, possesses the largest repertoir of myosins. Although the taxon sampling is very limited in this study, it is likely that the evolution of the arthropods was accompanied by taxon- and species-specific losses of certain myosin classes. *Daphnia *still contains a class-XIX myosin, that all other analyzed arthropods have lost, and four class-I myosins. Class-XIX myosins have also been found in Deuterostomia and Cnidaria. Also, all other arthropods have lost at least one of the class-I myosins. However, the remaining variants differ between the analyzed species, which means that they lost the class-I variants after separating from the next closest species. For example, *Apis *and *Nasonia *both have lost the class-I myosin variant C, that their closest relative *Pediculus *still has. *Pediculus*, however, specifically lost the variants B and D, respectively. All arthropods contain a non-muscle as well as a muscle myosin heavy chain gene (class-II myosins). The alternatively spliced muscle myosin heavy chain genes have been described elsewhere [32]. The *Drosophila *species and *Tribolium *have lost the class-3 myosin. The *Drosophila melanogaster *NinaC protein has previously been classified as a class-III myosin. Based on the analysis of more than 2000 myosins the NinaC protein does not group to the vertebrate class-III myosins and all arthropod homologs of NinaC have been grouped into a new class, class-XXI [7]. Surprisingly, *Nasonia *does not contain a class-VI myosin, that all other Metazoa contain, that have been analyzed so far,. The lack of the class-VI myosin might be a specific characteristic of *Nasonia vitripennis*, or due to sequencing and assembly problems, which are, however, unlikely given the high coverage of the *Nasonia *genome sequence. Finishing of the sequencing of the other two *Nasonia *genomes, which is in progress at the Baylor College of Medicine, will either confirm the lineage specific loss of the class-VI myosin or reveal sequencing problems of the *Nasonia vitripennis *genome. *Daphnia*, *Pediculus*, and *Apis *have lost the variant B of the class-VII myosin. The class-VII myosin, which they contain, is a clear homolog of the class-VII variant A myosins of the other arthropods. Another scenario would be, that the ancestor of *Apis*, *Nasonia*, and the clade containg the mosquito and *Drosophila *species has gained the class-VII myosin variant B via gene duplication of the variant A myosin. In this case, *Apis *specifically lost its class-VII myosin variant B. The *Drosophila *lineage has also completely lost the class-IX myosin. All arthropod genomes contain a class-XV, a class-XVIII, a class-XX, and a class-XXI myosin. The class-XXII myosin has independently been lost by several sub-lineages of the *Drosophila *species. The *Drosophila *species, that have been marked as having lost their class-XXII myosin, all still contain some of the exons of the ancient class-XXII myosin but spread over several hundred thousands of base pairs so that it is highly improbable that these pieces might belong to still functional genes.

**Figure 2:**
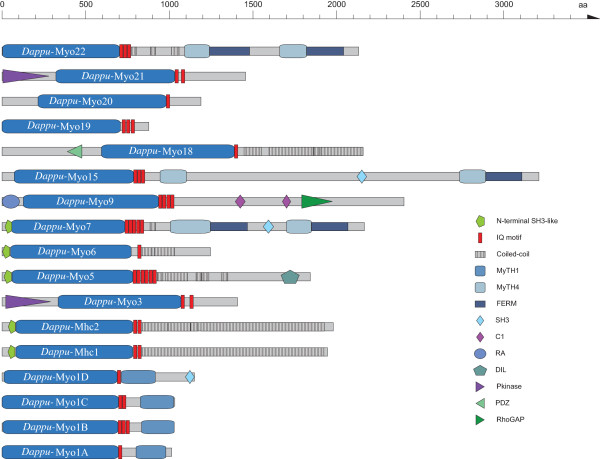
**Domain organisation of the *Daphnia pulex *myosins**. The sequence name is given in the motor domain of the respective myosin. A colour key to the domain names and symbols is given on the right except for the myosin domain that is coloured in blue. The abbreviations for the domains are: C1, Protein kinase C conserved region 1; DIL, *dilute*; FERM, band 4.1, ezrin, radixin, and moesin; IQ motif, isoleucine-glutamine motif; MyTH1, myosin tail homology 1; MyTH4, myosin tail homology 4; PDZ, PDZ domain; Pkinase, Protein kinase domain; RA, Ras association (RalGDS/AF-6) domain; RhoGAP, Rho GTPase-activating protein; SH3, *src *homology 3.

The domain organizations of the arthropod myosins are identical to those found for other members of the respective classes [7]. Figure 2 shows diagrams of the *Daphnia *myosins that have the largest diversity of the arthropod myosins. The class-XXI and the class-III myosins have an identical domain organization, although the phylogenetic analysis of their motor domains reveals two distinct classes. It is highly probable, that the class-XXI myosins are the result of an arthropod specific gene duplication of the ancient class-III myosin followed by the divergence of the new duplicate. The class-XXII myosins and the class-VII myosins have similar domain organizations. In contrast to the class-VII myosins, the class-XXII myosins lack the N-terminal SH3-like domain, they contain three instead of five IQ-motifs for the binding of calmodulin-like light chains, they have a longer coiled-coil regions containing domain till the first MyTH4 domain, and they lack the SH3 domain of the C-terminal tail.

### Analysis of the arthropod kinesins

**Figure 3:**
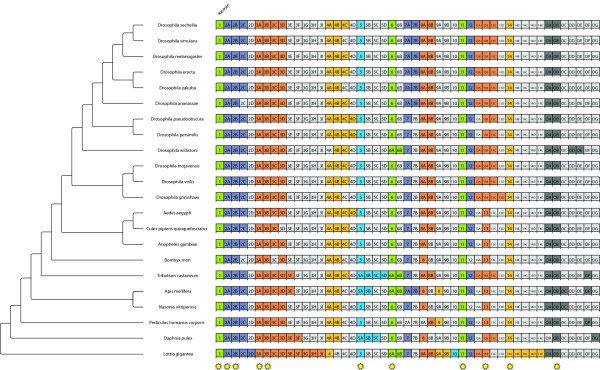
**Kinesin repertoire of the arthropods**. This chart shows the kinesin repertoire for all species in the analysis as in Figure 1.

For their classification, the kinesin motor domains have been used in a phylogenetic analysis together with the motor domains of the human kinesins [11,33]. The sequences have been named according to the standardized kinesin nomenclature [34] leaving some kinesins unclassified (Figure 3). Orphan kinesins, that are clear homologs, got the same variant designation to allow for better comparison. In general, all analyzed species contain species-specific sets of kinesins. Except for Drosophila pseudoobscura and *Drosophila persimilis*, which have identical sets of kinesins, even closely related species like the twelve analyzed *Drosophila *species have different kinesin inventories. Thus, it is likely that the evolution of the kinesin inventories of the analyzed arthropods is strongly determined by species-specific gene duplications and gene losses. Given the limited taxon and species sampling it is impossible to identify lineage-specific duplication and loss events. Some gene duplications and gene losses are especially interesting. In this respect, we will not consider the kinesin inventory of *Bombyx mori *because the genome has not been sequenced with high coverage and is highly fragmented. The *Drosophila ananassae *genome does not contain a kinesin-2C that all other arthropods have. *Drosophila willistoni *does not contain a kinesin-4A, but two class-VI kinesins and two species-specific kinesins that have not been classified yet, kinesin-D and kinesin-E. While most arthropods contain only one kinesin-5, *Tribolium *contains a set of four class-V kinesins. The *Pediculus *genome does not encode a class-VII kinesin, but encodes a kinesin-9 that is otherwise only found in *Apis*. None of the analyzed arthropods contains a kinesin-10. *Nasonia *does not contain a kinesin-12 that all other arthropods have. The set of class-XIII kinesins in the arthropods ranges from one to four homologs. *Tribolium*, *Apis*, *Nasonia*, *Pediculus*, and *Daphnia *contain one or two additional kinesins that could not be grouped to any of the known classes.

**Figure 4:**
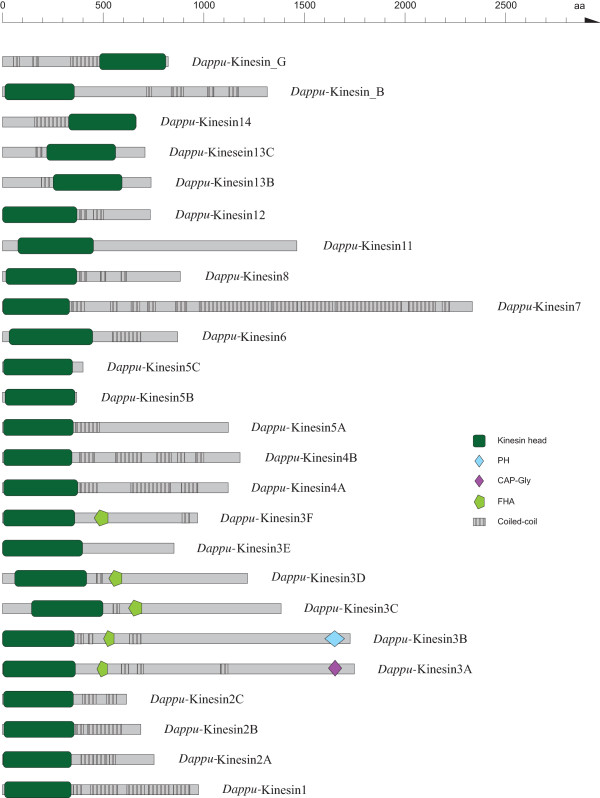
**Domain organisation of the *Daphnia pulex *kinesins**. The sequence name is given next to the respective kinesin. A colour key to the domain names and symbols is given on the right except for the kinesin domains that are coloured in dark-green. The abbreviations for the domains are: CAP-Gly, Cytoskeleton-associated protein-Gly; FHA, forkhead homology associated; PH, pleckstrin homology.

The *Daphnia *kinesins mainly consist of the kinesin motor domain and long coiled-coil regions in the tail (Figure 4). Only the class-III kinesins contain further domains that have been characterised and named. A characteristic of almost all class-III kinesins is an FHA domain following C-terminal to the motor domain. The class-III variant A kinesins also contain a CAP-Gly domain at the C-terminus, while the variant B kinesins contain a PH domain.

### The dynein/dynactin motor protein complex of the arthropods

**Figure 5:**
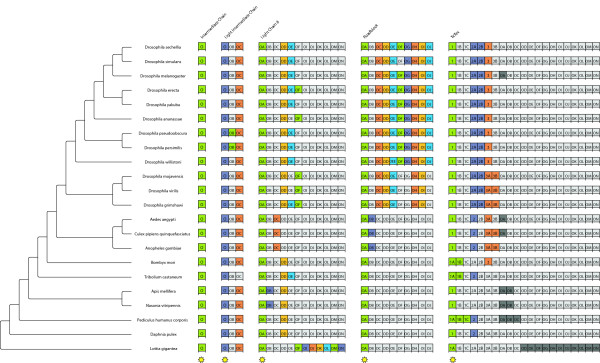
**Dynein repertoire of the arthropods**. This chart shows the dynein repertoire for all species in the analysis as in Figure 1. *

All arthropods contain members of all the cytoplasmic dynein subunits (Figure 5). The dynein heavy chain proteins belong to the longest proteins in eukaryotes having lengths of 3,500 to 5,000 amino acids. The genes of the dynein heavy chains have not been analysed and classified yet because their large size in combination with the high degree of fragmentation of many genomes render their complete identification and assembly impossible. All arthropods contain one intermediate chain. Except for *Tribolium*, the arthropods contain two light-intermediate chains. In addition, *Drosophila pseudoobscura *and *Drosophila persimilis *both contain a third light-intermediate chain. The sets of dynein light chains are very divergent in all analysed arthropods, although one of each of the LC8, Roadblock, and TcTex light chains is common to all species. In addition to these common light chains, all species have different numbers and variants of dynein light chains. It is remarkable that the *Drosophila *species have the largest number and most divergent set of light chains. Especially, they have five to eight additional light chains of the Roadblock family. The list of TcTex light chains also includes the ones that are associated with the axonemal dynein heavy chain. Because of their diversity it is not possible to specify, which of the TcTex light chains are associated with the cytoplasmic dynein heavy chain. Therefore, all TcTex homologs are listed.

**Figure 6:**
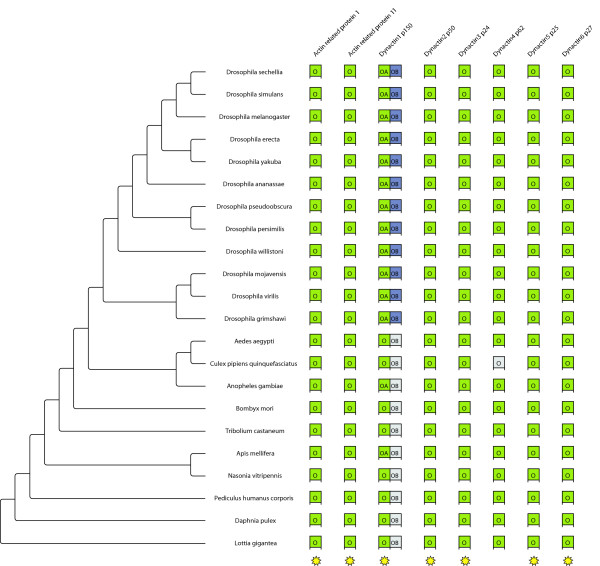
**Arp/Dynactin repertoire of the arthropods**. This chart shows the Arp/dynactin repertoire for all species in the analysis as in Figure 1.

Similar to the mammals, the arthropods contain one gene of each of the subunits of the dynactin complex (Figure 6). Only the genomes of the *Drosophila *species encode another version of the dynactin p150 subunit. These genes are close homologs to the well described Glued (dynactin p150) gene in *Drosophila melanogaster *[35] but have not been identified previously. We did not find any splice variants of any of the dynactin transcripts of the arthropods, although different splice forms exist for all of the mammalian dynactin subunits.

### Arthropod phylogeny

First, we calculated the phylogenetic tree of each of the protein families. When inspecting the phylogenetic tree of each protein family, it can be stated that three clades and their internal topologies are constant: The *Drosophila *clade, a clade of *Apis mellifera *and *Nasonia vitripennis*, and the clade of *Aedes aegypti*, *Culex pipiens quinquefasciatus*, and *Anopheles gambiae*. Only in the tree of the LC8 proteins (see Additional File 1), the clade of *Anopheles*, *Aedes *and *Culex *is placed within the *Drosophila *clade. All other species were placed at varying branches. The discrepancy among the phylogenetic trees based on the dynein and dynactin subunits was higher when compared to the ones based on myosins and kinesins (see Additional File 1). The trees calculated from myosins and kinesins only disagree in the positions of *Bombyx mori*, *Tribolium castaneum *and *Pediculus humanus corporis*.

**Figure 7:**
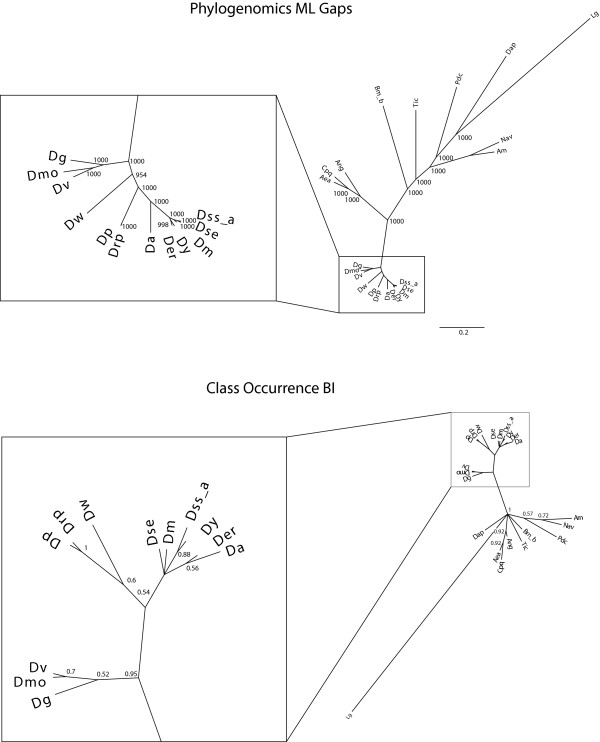
**Phylogenomics and Class Occurrence**. The trees illustrate the phylogenetic relationship between the arthropod species. The phylogenomic trees are based on a total of 682 concatenated protein sequences. Methods are indicated. The class occurrence tree was constructed using Bayesian inference based on the presence or absence of protein classes/variants as indicated in the inventory (Figures 1, 3, 5, and 6). The average standard deviation of split frequencies was 0.0087.

As has been found, when analysing the phylogeny of several homologs in a set of species, each homolog might result in a different phylogeny. This might result from the different rates of evolutionary change for different genes, from long-branch-attraction artifacts, or from sampling unrecognized paralogs [36]. Concerning unrecognized paralogs, we are confident that we were able to distinguish paralogs and orthologs since we have used very large datasets of protein sequences for the classification of the motor proteins with a wide taxonomic sampling ([7]; Kollmar, unpublished data). In order to compensate for this asynchronous evolution, a phylogenomics approach was used to infer the phylogeny of the 21 arthropods. For each protein family, the classes/variants, for which a homolog exists in every species, were concatenated resulting in more representative sequences by averaging out different rates of evolutionary change. For the dynein, dynactin and ARP (actin-related protein) proteins, only one of the homologs was found in all species, whereas eight of the myosins and ten of the kinesins are shared by all analysed arthropods (marked with stars in Figures 1, 3, 5, and 6). Thus, for each of the 22 species, 31 homologs were used, amounting to 682 motor protein sequences. The resulting trees are shown in Figure 7. Except for the placement of *Tribolium*, all four phylogenomics trees show identical phylogenies. All branches are supported by very high bootstrap values and are therefore reliable within the limits of the method. The placement of *Pediculus *depends on the method used. In the trees generated with neighbour joining (see Additional File 2), *Pediculus *forms a clade with *Nasonia *and *Apis*, whereas with maximum likelihood, only *Nasonia *and *Apis *are monophyletic and *Pediculus *is more closely related to *Daphnia*.

The phylogenetic tree inferred from the occurrence of classes/variants has a limited resolution and agrees only in some respects with the maximum likelihood tree: *Drosophila *form a clade, *Drosophila pseudoobscura *and *Drosophila persimilis *are monophyletic, *Drosophila virilis*, *Drosophila mojavensis *and *Drosphila grimshawi *are monophyletic and *Culex*, *Aedes *and *Anopheles *are monophyletic.

## Discussion

Most of the myosins that we discuss here have been identified and annotated in the course of the annotation of over 2000 myosins from more than 300 organisms [7]. Since then, the genome sequences of the arthropod species *Culex pipiens quinquefasciatus *and *Pediculus humanus corporis *have been finished as well as that of the mollusc *Lottia gigantea*, which we used as outgroup. All myosins have been grouped into 35 classes. The arthropods encode members of 13 of these classes, namely members of the classes I, II, III, V, VI, VII, IX, XV, XVIII, XIX, XX, XXI, and XXII. It has been found, that the *Drosophila melanogaster *NinaC protein, which has previously been classified as class-III myosin, is part of the new class-XXI [7]. Most arthropod genomes contain a real ortholog to the mammalian class-III myosins. Although both class-III and class-XXI myosins have an N-terminal kinase domain, the phylogenetic tree of the motor domain sequences clearly shows that both classes are distinct. *Daphnia pulex *contains the largest diversity of myosins, while the *Drosophila species *seem to have lost several classes, namely the members of class-III, class-IX, and class-XIX. Most of the *Drosophila species *have also lost their class-XXII myosin. Class-XXII myosins have two tandem repeats of MyTH4 and FERM domains like the class-VII myosin, but they miss the N-terminal SH3-like domain as well as the SH3 domain in the C-terminal tail. The specific function of a member of the class-XXII myosin has not been analyzed yet.

Of the kinesin superfamily the arthropods have members of all 14 specified classes [34] except for class-X. Class-IX kinesins have only been identified in *Apis mellifera *and *Pediculus humanus corporis*. However, the function of class-IX kinesins in not clear yet [11]. In addition to the kinesins, that could be classified, each of the analyzed arthropod species contains two or more kinesin homologs that could not be grouped to any of the known classes. Two of these orphan kinesins have been identified in all arthropod species except *Daphnia*, but some arthropods contain further species-specific kinesins. Notably, *Drosophila willistoni *contains two further kinesins, of which homologs have not been identified in any of the other sequenced arthropod genomes. Compared to the myosin repertoire, the kinesin inventory of the arthropods is far more varied. Although the analyzed arthropods have members of almost all classes, there are prominent differences in the subclass composition. Even the *Drosophila *species have different sets of kinesins. Thus, it is likely that the evolution of the kinesin diversity in arthropods is strongly determined by taxon- and species-specific gene losses and gene duplication events.

The arthropods contain a highly variable set of cytoplasmic dynein subunits. The dynein motor protein complex is build of dynein heavy chains, intermediate chains, light-intermediate chains, and the light chain 8, the Roadblock, and the TcTex light chains. All arthropods encode one dynein intermediate chain and a dynein light-intermediate chain. In addition, the closely related species *Drosophila pseudoobscura *and *Drosophila persimilis *contain another dynein light-intermediate chain. Of the light chains, the arthropods share one of each of the different types, the LC8, the Roadblock, and the TcTex light chains. All arthropods contain different numbers of further homologs of these light chains. Thus, they can build very specific cytoplasmic dynein complexes. For example, if all members of the Roadblock light chain family are also members of the cytoplasmic dynein complex the *Drosophila *species could build up to nine different cytoplasmic dynein complexes just by exchanging light chains of the Roadblock family. These different Roadblock light chains might bind different cargoes and by tissue specific or developmentally regulated expression of these Roadblock genes the *Drosophila *species might be able to fine tune their dynein mediated transport processes. Thus, there are far more possibilities to adjust cargo binding by combining different light chains than by using the dynein activator complex, dynactin. The arthropods contain one of each of the eleven dynactin subunits. Alternative splice forms have not been identified. Only the *Drosophila *species contain a further homolog of the p150 (Glued) subunit, that has not been identified and characterized yet.

It has been observed, given heterogeneous evolutionary rates, that the results of the maximum likelihood method are statistically more robust than the ones produced by neighbour joining [37]. Therefore we conclude that *Apis*, *Nasonia*, and *Pediculus *are not monophyletic, but that *Pediculus *is more closely related to *Daphnia*. The class occurrence tree shows that the classification system we used for the protein families does not contradict the finding of the sequence-based phylogenetic inference.

Our study suggests the following phylogeny: The *Drosophila *clade is composed of the *Drosophila simulans*/*Drosophila sechella *clade which forms a clade with *Drosophila melanogaster*. This clade together with the *Drosophila yakuba*/*Drosophila erecta *clade forms the melanogaster subgroup. This subgroup together with *Drosophila ananassae *forms the melanogaster group. The melanogaster group is most closely related to the obscura group, a clade that consists of *Drosophila pseudoobscura *and *Drosophila persimilis*. The closest relative to the obscura group is *Drosophila willistoni*. All of the before mentioned species form the subgenus Sophophora. Its sister subgenus is Drosophila, consisting of the clade of *Drosophila virilis*/*Drosophila mojavensis *and *Drosophila grimshawi *(taxonomy as in [29]). The phylogeny of the *Drosophila *clade is in exact agreement with what has been found in an analysis based on the complete genome sequences of the twelve species [29].

The closest relatives to the *Drosophila *clade are *Aedes aegypti *and *Culex pipiens*, forming one clade, and *Anopheles gambiae*. All these species belong to the Diptera. The placing of the remaining species, that have been analyzed here, is mainly in accordance with an analysis of 128 arthropod species that was based on 275 morphological variables as well as 18S and 28S rDNA data [38]. In accordance with this study, the Lepidoptera, to which *Bombyx mori *belongs, are the closest relatives to the Diptera forming the Mecopteroidea. Also in aggreement with the morphological data, the Hymenoptera (*Nasonia vitripennis*/*Apis mellifera*) are basal to the Mecopteroidea together forming the Holometabola, and the Phthiraptera (*Pediculus humanus corporis*) are basal to the Holometabola. The main difference between our study and the analysis of the morphological data is the placement of *Tribolium castaneum*, a Coleoptera species. Our study placed *Tribolium *closer to the Mecopteroidea while the other study placed the Coleoptera outside the Hymenoptera and Mecopteroidea. *Daphnia pulex*, a Crustacea species, diverged earlier to all the other Hexapoda species.

## Conclusion

In this analysis, we were able to resolve the phylogenetic relationship of 21 completely sequenced arthropod species based in their motor proteins. A large number of sequences were used that have been checked manually. We have systematically analyzed the protein inventory of all species as well as the domain composition of all members of the four protein families in *Daphnia pulex*. When inferring phylogenetic trees from the sequence data, variations in evolutionary speed were accounted for by using a phylogenomics approach. This analysis produced a phylogenetic tree that is highly resolved and that has statistically well supported branchings. Our findings are in accordance with results from studies based on whole genome and rDNA sequences as well as morphological variables. We can conclude that from all arthropods analyzed, *Daphnia pulex *is the most basal one. *Pediculus humanus corporis *is the closest relative to *Daphnia*, followed by the clade of *Apis mellifera *and *Nasonia vitripennis*. Next, *Tribolium castaneum *and *Bombyx mori *diverged, followed by the mosquito species and the Drosophila clade.

## Methods

### Identification and annotation of the arthropod myosins, kinesins, and dynein/dynactin subunits

The genes for *Aea*, *Ang*, *Am*, *Bm*, *Cpq*, *Da*, *Der*, *Dg*, *Dm, Dmo*, *Drp*, *Dp*, *Dse*, *Dss*, *Dv*, *Dy*, *Dw*, *Nav*, *Pdc*, and *Tic *have been obtained by TBLASTN searches against the insects section of the NCBI wgs database [39]. The *Dap *sequences have been obtained by TBLASTN searches against the 8.7× coverage Dappu v1.1 draft genome sequence assembly (September, 2006) provided by the DOE Joint Genome Institute [40] and the *Daphnia *Genomics Consortium [41]. All hits were manually analysed at the genomic DNA level. The correct coding sequences were identified with the help of the multiple sequence alignments of the corresponding proteins. In this process, the sequence alignments of all proteins contained in our in-house version of CyMoBase have been used. As the amount of protein sequences increased (especially the number of sequences in classes with few representatives), many of the initially predicted sequences were reanalysed to correctly identify all exon borders. Where possible, EST data available from the NCBI EST database has been analysed to help in the annotation process. All sequence related data (names, corresponding species, GenBank ID's, alternative names, corresponding publications, domain predictions, and sequences) and references to genome sequencing centers are available through the CyMoBase [42,43].

### Building trees

The phylogenetic trees based on protein sequences were generated using two different methods: 1. Neighbour joining using the GONNET substitution matrix with bootstrapping (1,000 replicates) using ClustalW 2.0 [44]. 2. Maximum likelihood (ML) [45] using a JTT model with estimated proportion of invariable sites and bootstrapping (1,000 replicates) using PHYML [46].

The sequence data, which was used for the analyses, were multiple sequence alignments consisting either of single homologous sequences from each species or multiple concatenated homologous sequences from each species (phylogenomics approach). For comparison, multiple sequence alignments were used including columns with gaps or with columns containing gaps removed.

The class occurrence tree was generated using Bayesian inference with a binary model using MrBayes 3.1.2 [47]. For each species the existence/non-existence of a protein class/variant was used as a binary character as depicted in Figure 7. Using this encoding, each species is represented by a series of binary characters, one for each protein class/variant. Constant rates were used whereas gamma-distributed rates gave very similar results. The tree was generated using 1.000.000 generations and a burnin of 500.000 generations since at that point the average standard deviation of split frequencies fell below 0.011.

### Domain and motif prediction

Protein domains were predicted using the SMART [48,49] and Pfam [50,51] web server. The prediction of protein motifs (coiled coils, leucine zipper, etc.) is mainly based on the results of the predict-protein server [52,53]. The IQ-motifs and N-terminal domains of the myosins were predicted manually based on the homology to similar domains of other myosins included in the multiple sequence alignment of the myosins. The recognition motifs included in the SMART and Pfam databases are too restrictive, as the motifs have been created based on the small datasets available some years ago.

## Authors' contributions

FO performed the data analysis of the myosins, kinesins, and dynein subunits, as well as the phylogenetic analysis of all sequences. SB assembled all dynactin sequences and performed their analysis. MK assembled all myosin, kinesin, and dynein sequences. All authors wrote and approved the final manuscript.

## Supplementary Material

Additional file 1 **Phylogenetic trees of the motor proteins**. The file contains the phylogenetic trees of the concatenated sequences of the myosin, the kinesins, the dynein subunits, the dynactin subunits, and the ARP proteins. Click here for file

Additional file 2 **Phylogenetic tree of the arthropods based on the neighbor joining method**. The file contains the phylogenetic tree of the concatenated sequences of all motor proteins calculated using the neighbor joining method. Click here for file
