# Supplementary material for: Reconstructing the phylogeny of 21 completely sequenced arthropod species based on their motor proteins
Source: BMC Genomics. 2009 Apr 21;10:173. doi: 10.1186/1471-2164-10-173 (PMC2674883; doi:10.1186/1471-2164-10-173)

Myosin

	Full Sequence	Head Sequence

NJ / Gaps

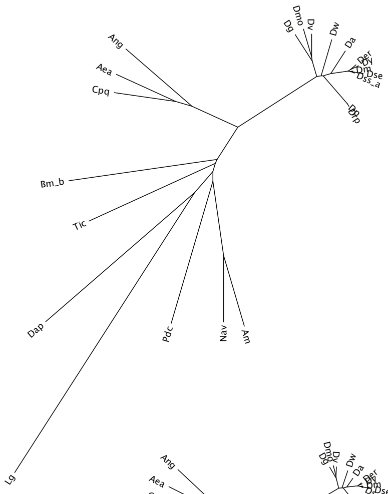

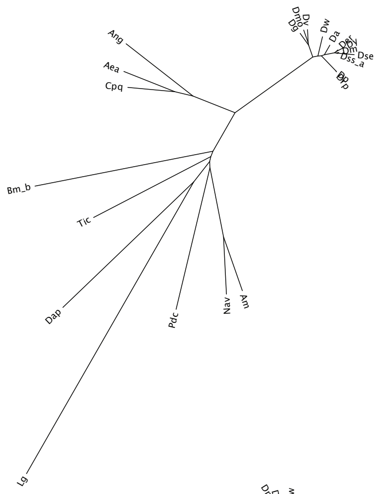

NJ / No Gaps

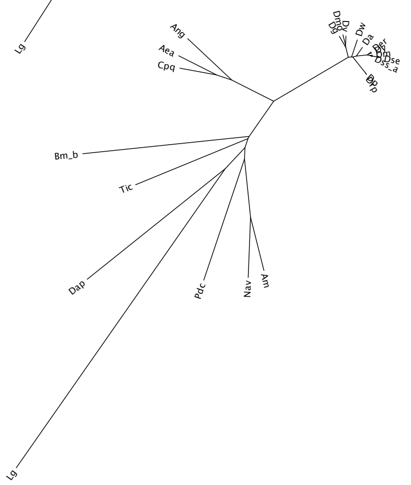

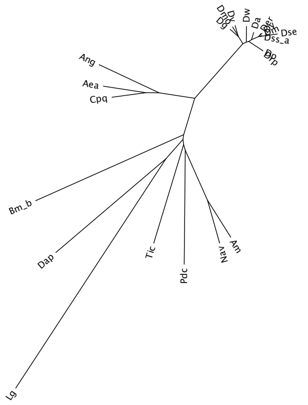

ML / Gaps

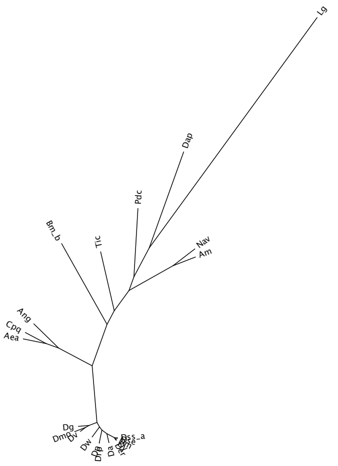

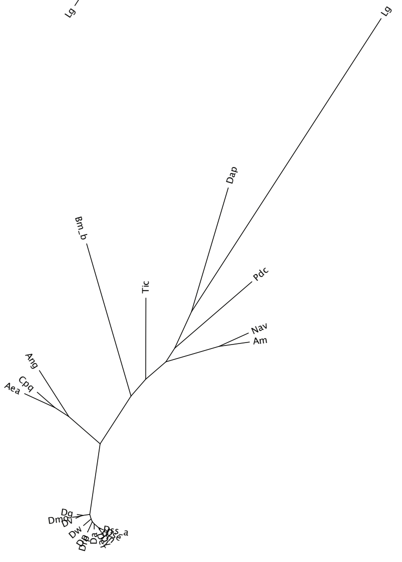

ML / No Gaps

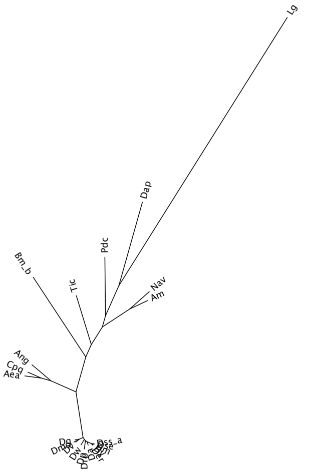

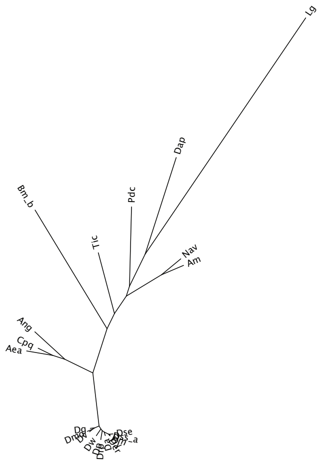

Kinesin

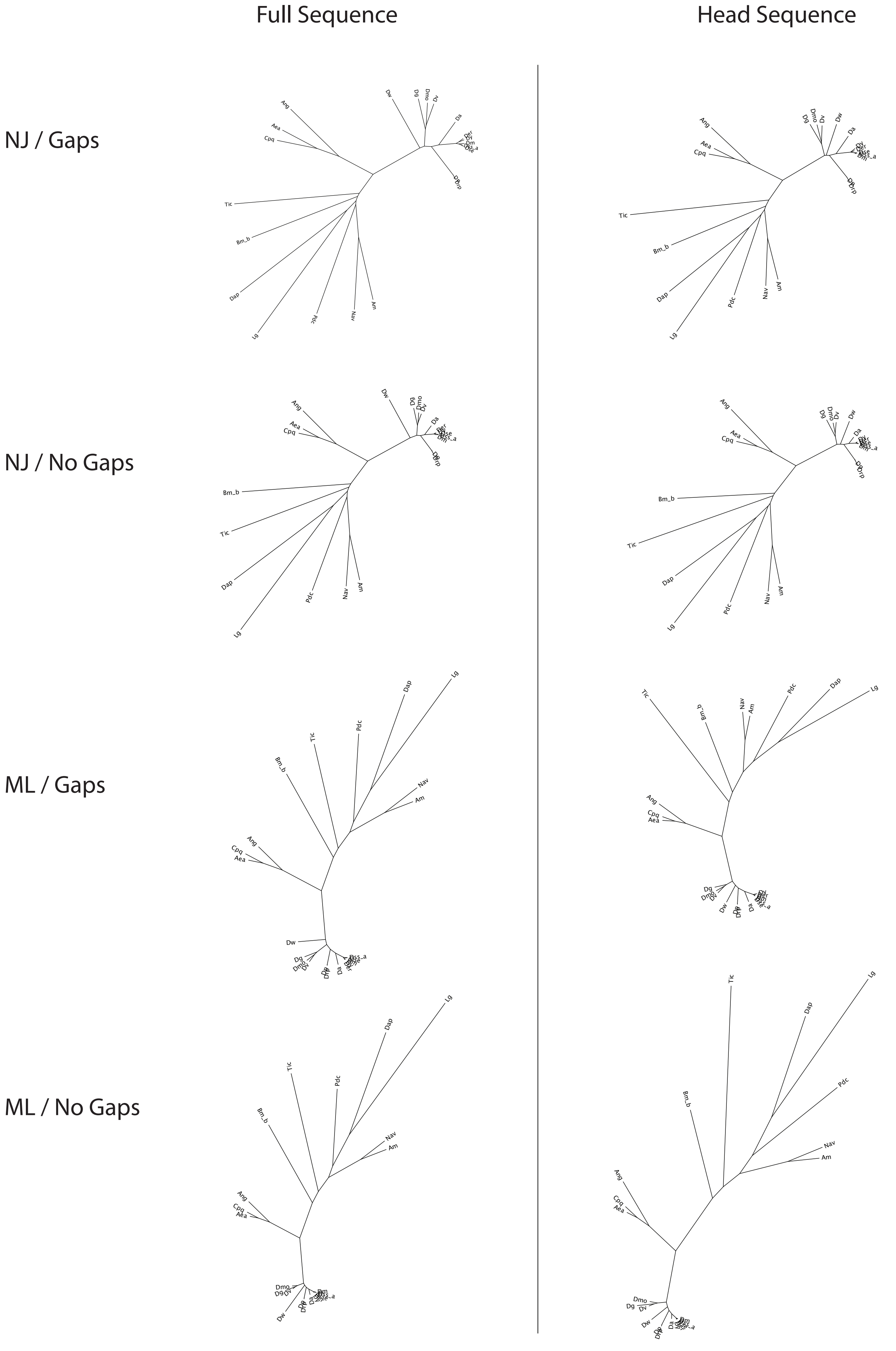
Full Sequence
Head Sequence
NJ / Gaps
NJ / No Gaps
ML / Gaps
ML / No Gaps
Ang
Aea
Cpq
Tic
Bm_b
Dap
Lg
Pdc
Nav
Am
Dw
Dg
Dmo
Dv
Da

Dynein

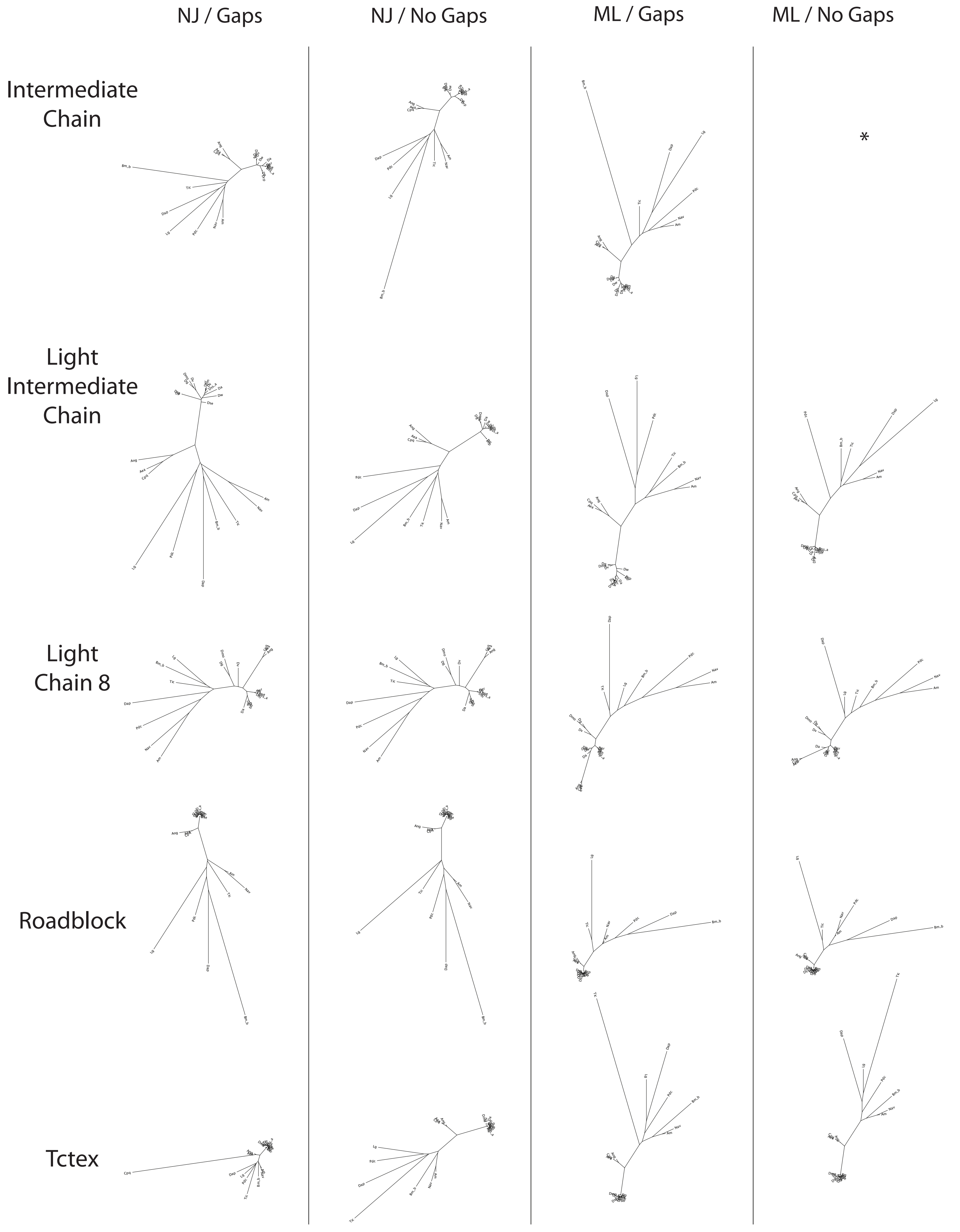
NJ / Gaps
NJ / No Gaps
ML / Gaps
ML / No Gaps
Intermediate Chain
*
Light Intermediate Chain
Light Chain 8
Roadblock
Tctex

Dynactin 2

	NJ / Gaps	NJ / No Gaps	ML / Gaps	ML / No Gaps
Dynactin3 p24				
Dynactin5 p25				
Dynactin6 p27				

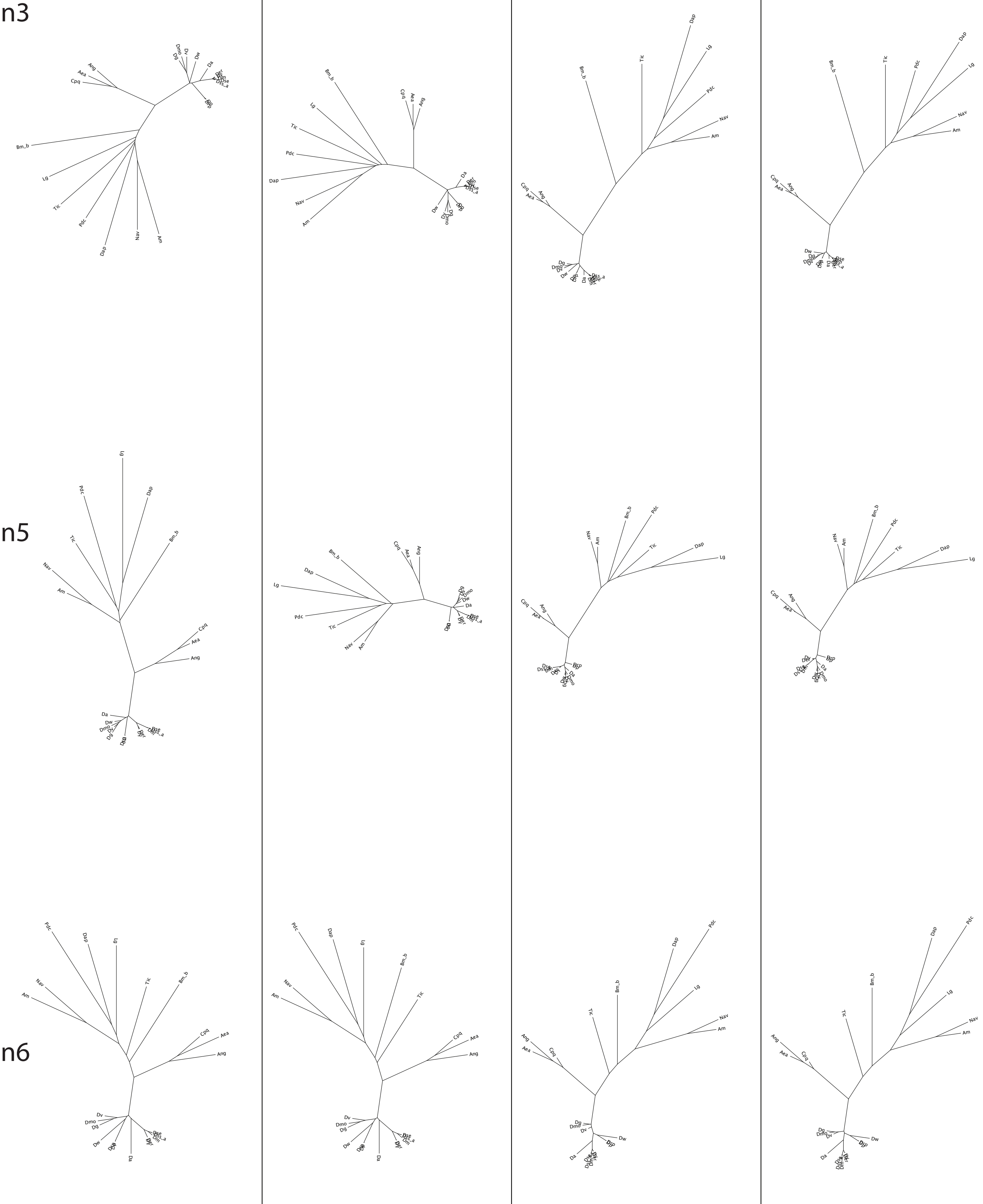

Arp & Dynactin 1

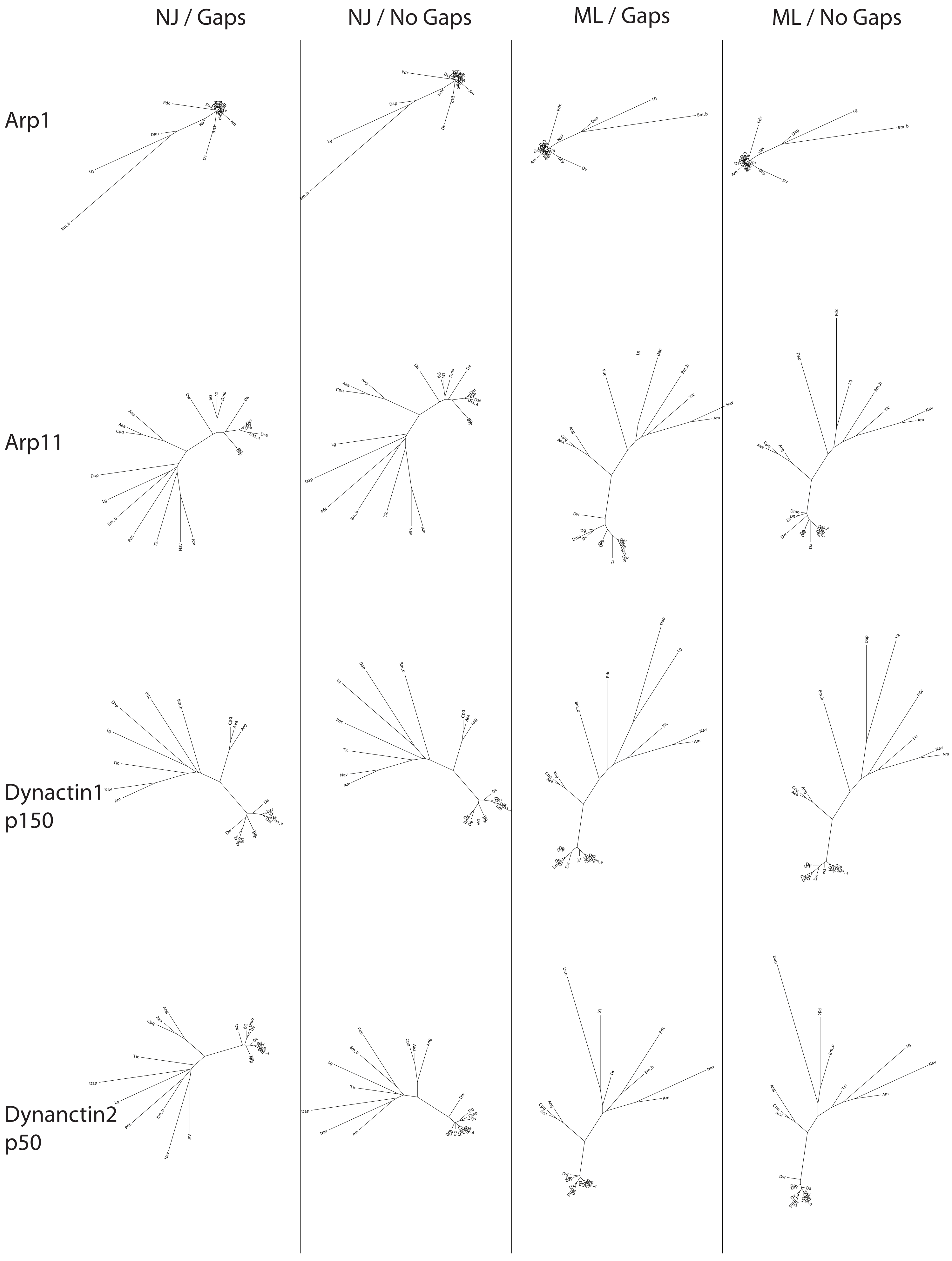

Supplement: Additional file 1 — Phylogenetic trees of the motor proteins. The file contains the phylogenetic trees of the concatenated sequences of the myosin, the kinesins, the dynein subunits, the dynactin subunits, and the ARP proteins. [file 1471-2164-10-173-S1.pdf]
