# Supplementary figures and images for: Reconstructing the phylogeny of 21 completely sequenced arthropod species based on their motor proteins
Source: BMC Genomics. 2009 Apr 21;10:173. doi: 10.1186/1471-2164-10-173 (PMC2674883; doi:10.1186/1471-2164-10-173)

# Phylogenomics NJ Gaps

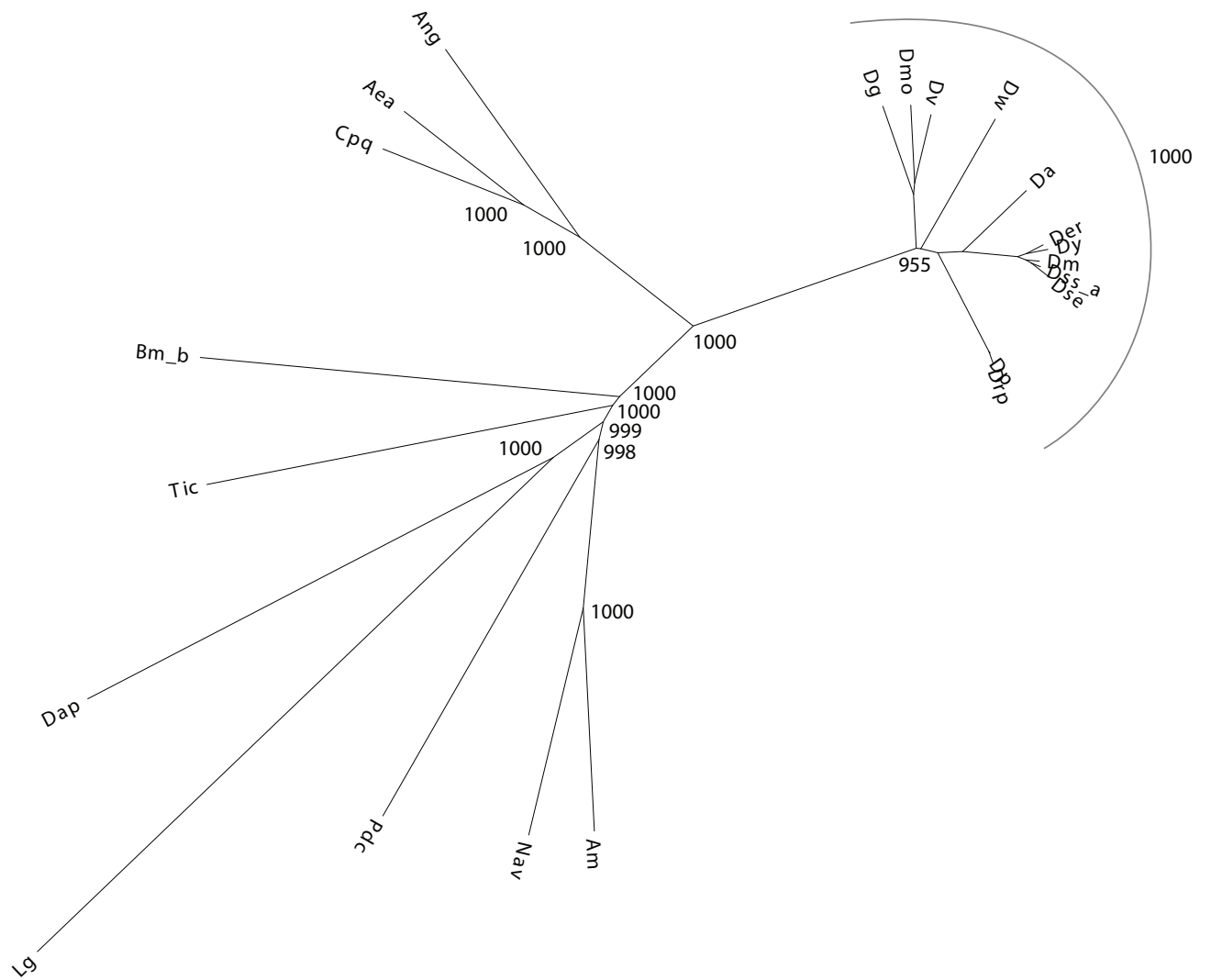

Supplement: Additional file 2 — Phylogenetic tree of the arthropods based on the neighbor joining method. The file contains the phylogenetic tree of the concatenated sequences of all motor proteins calculated using the neighbor joining method. [file 1471-2164-10-173-S2.pdf]
